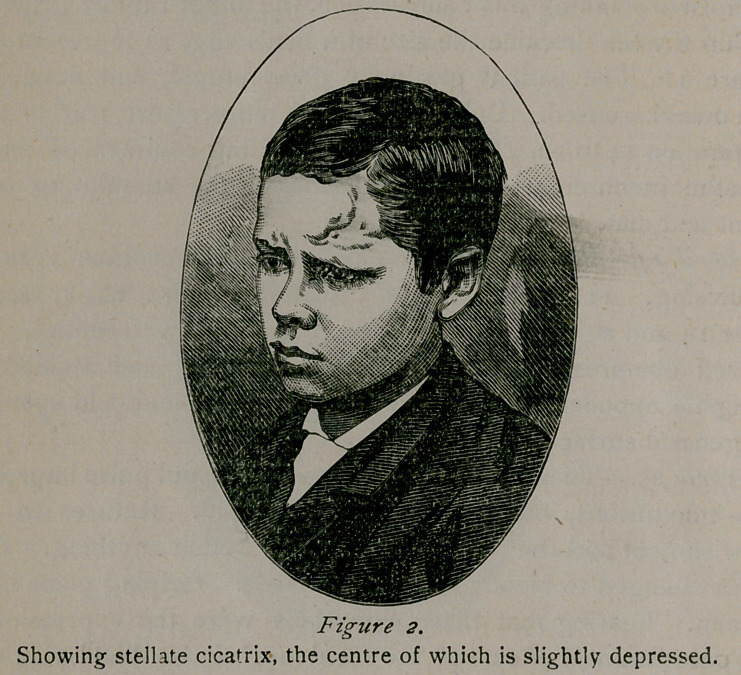# A Case of Compound Comminuted and Depressed Fracture of the Skull—Trephining—Fungus—Recovery

**Published:** 1886-02

**Authors:** Howard J. Williams

**Affiliations:** Macon, Ga.


### A CASE OF COMPOUND COMMINUTED AND DEPRESSED FRACTURE OF THE SKULL—TREPHINING—FUNGUS—RECOVERY.

BY HOWARD J. WILLIAMS, M. D., MACON, GA.

W. S., white, aged 13 years, while hunting, on the afternoon of April 2, 1885, attempted to discharge an overloaded gun, which exploded, fracturing his skull and injuring him slightly otherwise. After the gun exploded he ran forwards about fifteen feet and fell upon his face. He was not stunned, and when picked up was able to walk, with assistance, some distance. He was brought to the city in a wagon, and I was called to see him about 6 p. m.

The wound in the soft parts extended from half an inch above the internal angle of the left eye-brow upwards and outwards two inches to the margin of the hair. A probe passed into the wound discovered that the bone was depressed. The pulse was good; breathing regular; pupils moderately dilated, but responded to light; mind unimpaired; phonation good. He had not lost much blood, nor was he suffering from shock to any great degree.

I administered a hypodermic of morphine (gr. ¼), cleansed the wound and ordered that he should be kept perfectly quiet, and cloths wet with ice-water to be kept constantly on the wound during the night. There being no urgent symptoms, I decided to wait until morning before adopting further treatment.

*April 3., 9 a. m.*—Pulse 100, full and strong; temperature 100.2°; breathing good ; pupils sluggish ; slight delirium during the night, but rational when I saw him. Examining the wound again with the probe, I was convinced that the bone was comminuted, and that as it was a compound comminuted and depressed fracture, trephining was indicated. I so stated to the parents, and told them that I would bring with me in the afternoon another physician, and, if necessary, we would then operate.

7 p. m., accompanied by Dr. McHatton, I returned. The boy had vomited twice since I saw him. Pulse 108, full and strong; temperature 100.2°; respirations 14, and sighing; mind clear, but answers slowly, and disposed to be left alone; pupils slightly contracted and react sluggishly. We agreed that trephining was indicated, but for want of sufficient light decided to postpone until early next morning. Morphia, gr. 1-6, p. r. n., and cold applications to be continued.

*April 4, 7 a. m.*—Patient still in the same condition. Everything being in readiness, Dr. McHatton administered ether. I enlarged the external wound by a crucial incision, and, peeling up the periosteum, the bone was found to be comminuted over an area of one inch and depressed into the brain matter from one-half to three-quarters of an inch. There had been an escape of a small amount of brain matter. An attempt was made to raise and remove the fragments of bone, but that being impossible, I applied the trephine. The fragments were then removed and a depressed angle of bone raised with the elevator. The wound was thoroughly cleansed, the membranes carefully replaced and the external wound closed with sutures, except the lower angle, which was left for drainage. A compress, wet with a 1 to 1,000 parts solution bichloride of mercury, was applied to the wound and retained by a roller bandage. This dressing was to be kept constantly wet with the above solution, morphia to be used as needed, and the diet to be light.

6 p. m., pulse 88; temperature 98.6°; respiration 16; mind clear; some vomiting.

*April 5.*—Some wandering during the night; vomiting ceased; pulse 90; morning temperature 99.2°, evening, 100.2°; respirations, 13 to 12 and sighing.

*Figure 1.*
Showing fungus size of hen's egg, dark spots red—white suppurating points.

The patient from this date progressed very well. The pulse and respirations became normal, and the temperature ranged between 98 and 100.8°. There were no serious symptoms except some vomiting, headache and photophobia. The wound united well except the lower part, which continued to discharge.

*April 14.*—Dressing the wound to-day, I noticed that the edges, which for two days had been bulging, were separating and a fungus mass protruding. This mass bled freely, was painless, elastic, pulsated synchronously with the brain, and discharged a thin, inoffensive pus. His general condition was good and his mind clear. A tonic treatment of iron, quinine and whisky, anodyne, doses of morphine as needed, and a highly nutritious diet was commenced. Systematic compression of the mass was effected by means of compressed sponges held firmly on the wound by roller bandages, and all this to be kept wet with the antiseptic solution to cause expansion of the sponges.

Notwithstanding this compression, the tumor rapidly grew and within a week became the size of a hen's egg, as represented in figure 1. The patient gradually grew stupid, and noticed no one unless aroused. Pulse 100 to 116, temperature 100° to 101°, respiration 14 to 12. Diarrhœa and vomiting commenced, and he became much emaciated, notwithstanding the stimulating treatment and diet.

*April 24.*—Patient comatose and general condition very unpromising. 11:30 p. m., pulse 130, temperature 104.4, respirations 14 and sighing. Convulsive twitching of extremities. Removed compressed sponges from the wound and applied ice. Morphia hypodermically, inunctions of quinine and cold sponging of general surface.

*April 25.*—Temperature 98.6, respirations and pulse improved. No convulsions, but mind still very stupid. Refuses to take nourishment and the stomach unable to retain anything. Diarrhœa changed to bloody dysenteric stools. Griping pains in abdomen. Fearing that these symptoms were the expressions of the constitutional effects of the local use of the bichloride solution, I discontinued it.

*April 28.*—The boy's condition unimproved, except that the blood had disappeared from the stools and the diarrhœa had gradually ceased. Pulse 100 to 140, temperature 99° to 100°, respirations 8 to 12. His stomach was too irritable to retain food or medicines; his bowels, however, were sufficiently quiet to begin to employ enemas of beef tea.

*April 29.*—Pulse 100, temperature 99.6°, respirations 12. Mind flighty. Vomiting less frequent. As he asked several times for buttermilk, and refused other nourishment, I ordered small quantities to be given to him at frequent intervals, hoping that it might settle his stomach.

Soon after commencing the buttermilk, the stomach became quiet and his appetite, strength and flesh began to return. The tumor had ceased to grow, but was perforated at its base by many sinuses, which discharged copiously and permitted a probe to pass through and through the mass.

*May 4.*—Through the sinuses in the tumor I passed linen cords,

*Figure 2.*

Showing stellate cicatrix, the centre of which is slightly depressed.

which I tied together tightly, hoping to strangulate the growth and thus cause its separation. This had no effect, and after a few days' trial I abandoned it, deciding to wait until my patient had regained sufficient strength, and then resort to the knife and try compression once more.

*May 27.*—Patient up and about; tumor still the size of a hen's egg. Removed the tumor without an anæsthetic; considerable pain and hemorrhage attending the operation. Compression with compressed sponges again employed.

The boy was confined to his bed only eight days and his general condition remained good. The fungus, however, rapidly recurred and within a month was the size of a small orange.

*June 22.*—Painted the surface of the tumor with a pencil of nitrate of silver and applied rubber adhesion plaster to compress growth. One end of a narrow strip of plaster (half an inch wide and several inches long) was attached to the skin at the base of the tumor, and then the strip was wound very tightly around it in a spiral direction until the entire mass was covered. Over all a bandage was placed to protect the dressing.

This dressing was repeated every other day; each time a thin slough come away. At the end of a month the fungus was reduced to the size of a chestnut.

*July 25.*—Applied around the base a narrow rubber band to strangulate the mass. At the end of the third day it was so thoroughly deadened that I cut it away without pain or hemorrhage. I then applied to the stump iodoform and zinc ointment; healthy granulations sprung up and within two weeks the wound had healed.

At the present time, November 5, the boy is in fine health, is going to school, and presents appearance as shown in figure 2. There seems to be no trouble with his brain, no headaches or other unpleasant symptoms, and his mental faculties are apparently as good as any other boy of his age and circumstances.

## Figures and Tables

**Figure. 1. f1:**
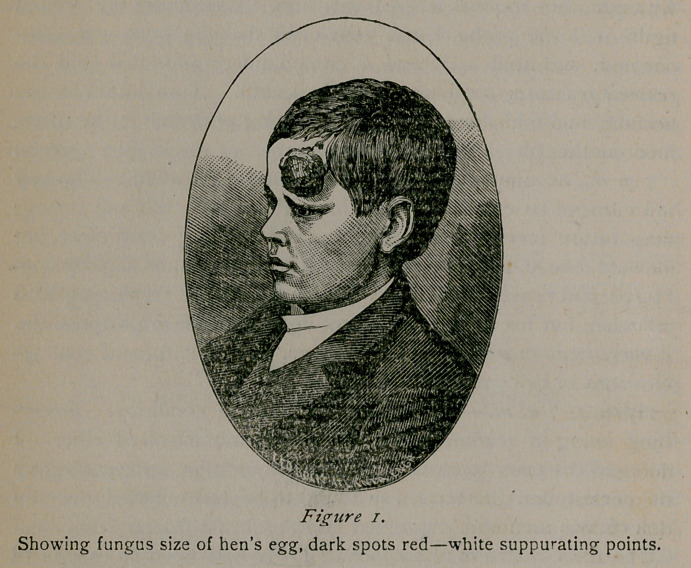


**Figure. 2. f2:**